# Correlation between Neck Circumference and Other Anthropometric Measurements in Eight Latin American Countries. Results from ELANS Study

**DOI:** 10.3390/ijerph182211975

**Published:** 2021-11-15

**Authors:** Reyna Liria-Domínguez, Marcela Pérez-Albela, María-Paz Vásquez, Georgina Gómez, Irina Kovalskys, Mauro Fisberg, Lilia Yadira Cortés, Martha Cecilia Yépez García, Marianella Herrera-Cuenca, Attilio Rigotti, Gerson Ferrari, Rossina G. Pareja, on behalf of the ELANS Study Group

**Affiliations:** 1 Instituto de Investigación Nutricional, Lima 15026, Peru; rpareja@iin.sld.pe; 2 Facultad de Ciencias de la Salud, Carrera de Nutrición y Dietética, Universidad Peruana de Ciencias Aplicadas-UPC, Lima 15067, Peru; marcela.par31@gmail.com (M.P.-A.); mariapazvp07@gmail.com (M.-P.V.); 3 Departamento de Bioquímica, Escuela de Medicina, Universidad de Costa Rica, San José 11501-2060, Costa Rica; georgina.gomez@ucr.ac.cr; 4 Carrera de Nutrición, Facultad de Ciencias Médicas, Pontificia Universidad Católica Argentina, Buenos Aires C1107 AAZ, Argentina; ikovalskys@gmail.com; 5 Centro de Excelencia em Nutrição e Dificuldades Alimentaes (CENDA), Instituto Pensi, Fundação José Luiz Egydio Setubal, Hospital Infantil Sabará, São Paulo 01228-200, Brazil; mauro.fisberg@gmail.com; 6 Departamento de Pediatria, Universidade Federal de São Paulo, São Paulo 04023-061, Brazil; 7 Departamento de Nutrición y Bioquímica, Pontificia Universidad Javeriana, Bogotá 110231, Colombia; ycortes@javeriana.edu.co; 8 Colegio de Ciencias de la Salud, Universidad San Francisco de Quito, Quito 17-1200-841, Ecuador; myepez@usfq.edu.ec; 9 Centro de Estudios del Desarrollo, Universidad Central de Venezuela (CENDES-UCV)/Fundación Bengoa, Caracas 1053, Venezuela; manyma@gmail.com; 10 Centro de Nutrición Molecular y Enfermedades Crónicas, Departamento de Nutrición, Diabetes y Metabolismo, Escuela de Medicina, Pontificia Universidad Católica, Santiago 8330024, Chile; arigotti@med.puc.cl; 11 Escuela de Ciencias de la Actividad Física, el Deporte y la Salud, Universidad de Santiago de Chile (USACH), Santiago 7500618, Chile; gerson.demoraes@usach.cl

**Keywords:** waist circumference, body mass index, neck circumference, obesity, overweight

## Abstract

Neck circumference (NC) is being used to identify the risk of chronic diseases. There is a high prevalence of overweight and obesity in Latin America, and neck circumference is a simple and practical measurement to assess this, especially in primary health centers. We analyzed the correlation between the NC anthropometric indicator and other anthropometric measurements such as BMI and waist circumference (WC) in eight Latin American cities. We applied Pearson’s correlation to identify the correlate NC with the other anthropometric variables stratified by sex; the sensitivity (Se) and specificity (Sp) by sex were evaluated according to the cut-off established with the Youden Index. The strongest correlations between NC and WC were found when stratified by sex (women: r = 0.71; men: r = 0.69, respectively) followed by the correlation between NC and BMI (r = 0.65, both sex). NC cut-off points of 39.0 cm in men and 32.9 cm in women identified those individuals with an increased WC and 39.8 and 33.7 cm, respectively, for a substantial increase in WC. For BMI ≥ 25.0 kg/m^2^ for men, the cut-off point was 37.5 cm, and for women, it was 33.1 cm, and for BMI ≥ 30 kg/m^2^, the cut-off points were 39.2 and 34.2 cm, for men and women, respectively. Conclusion: NC proved to be a useful, practical, and inexpensive tool that can be used to identify, evaluate, and monitor overweight and obese individuals.

## 1. Introduction

Excessive fat accumulation in the body is detrimental to health, as it is associated with different diseases such as type 2 diabetes mellitus, hypertension, and an increased risk of cardiovascular disease [1,2].

There are different methods or indicators to measure excess fat. Among the most common are body mass index (BMI), waist circumference (WC), waist/hip ratio, waist/height, and skinfolds. BMI is the simplest and best-known method of all. However, it has limitations, since it does not measure body fat or its location, nor can it differentiate between fat and lean tissue, resulting, for example, in a diagnosis of overweight or obesity in people with a high percentage of muscle mass [3]. Another important and commonly used marker is WC, which is widely used to determine the accumulation of abdominal fat [4,5]. WC is more specific than BMI for the determination of metabolic risk, as it distinguishes body fat, specifically visceral fat located at the abdominal level, from lean mass [4,6,7]. However, in the case of WC, the population, especially women, may feel discomfort when being measured and may be affected by the time of day when it is measured [8].

Fat can accumulate in different areas of the body, and the location can affect the degree of risk of illness. Fat accumulated in the upper part of the body (e.g., neck fat) can be related to a higher risk than fat accumulated in the abdomen [9]. This can be because fat accumulated in the neck is located in a separated compartment, in contrast to abdominal fat, which is divided into subcutaneous and intra-abdominal deposits [10]. Moreover, neck circumference (NC) has been shown to be correlated with risk factors independently of BMI and WC [9]. It has been associated with the components of metabolic syndrome such as elevated blood pressure, hypertriglyceridemia, hypercholesterolemia, elevated fasting glucose, insulin resistance [9], and obstructive sleep apnea [10,11].

NC could be a good indicator of cardiovascular risk, and may be an alternative tool, specifically for the measurement of upper subcutaneous fat. It is an anthropometric measurement characterized by its simplicity and practicality and it is not affected by the time of day when it is measured [12]. WC, however, is sensitive to abdominal distension and can be affected by gas exchange on inhalation and exhalation [13]. BMI is a universal measure practiced by health personal, but it does not reflect the body fat composition [14].

Latin America has high rates of excess weight and chronic diseases that require attention, especially in urban areas. First-level health facilities tend to have a high demand for work, and a simple and practical measure to carry out can help identify populations at risk of metabolic diseases. Our interest was to evaluate if there is a correlation between NC with BMI and WC in a Latin American population living in urban areas and to establish cut-off points where NC reaches the highest sensitivity and specificity to determine overweight and obesity. This measure could be used in different epidemiological studies or in the clinical area as a reliable, simple, fast, low-cost, and less invasive way than other anthropometric measurements, especially those involving skinfold and WC measurements.

## 2. Materials and Methods

### 2.1. Study Design

This was a secondary analytical study using information from the ELANS database. The ELANS study is a cross-sectional, multicenter nutritional and health study (2014–2015), conducted in urban populations from eight Latin American countries (Argentina, Brazil, Chile, Colombia, Costa Rica, Ecuador, Peru, and Venezuela) [15].

### 2.2. Population

We included people aged 15 to 65 years from more populated urban areas and excluded pregnant and lactating women (in the first 6 months postpartum), people with physical disabilities that may affect food intake and/or physical activity, people living in a residential setting other than a home (hospitals, regiments, and nursing homes), and those who cannot read. In our study, we mainly excluded people with incomplete information for the variables of interest.

### 2.3. Study Sample and Sampling

This study included the data analysis of 9218 participants. The Epidat program was used to calculate the statistical power. Considering a correlation coefficient of 0.50 and a confidence level of 95%, the sampling design was complex and multistage stratified by sex, age, and socioeconomic status. Details of the sampling are presented in the study publication [15].

### 2.4. Data Collection Procedure

Sociodemographic: A questionnaire was used to collect information on demographic data: age, sex, years of education, and marital status. In addition, socioeconomic status was also assessed by means of a questionnaire and classified into three strata (high, medium, and low) according to the national indexes used in each country [15].

Anthropometry: All subjects were weighted and measured with the least possible clothing. Measurements were taken in the following order: weight, height, WC, and NC. All measurements were made according to WHO [16] and ISAK (WC and NC) standards [17]. For weight, a Seca Model 813 electronic scale was used, previously calibrated, with a capacity of up to 200 kg and an accuracy of 0.1 kg. The participant was asked to step on the scale with the least clothing possible. To measure height, a Seca Model 213 portable stadiometer was used, with a capacity of 205 cm and an accuracy of 0.1 cm. For circumference measurements, a Seca Model 201 retractable measuring tape was used, with a capacity of 205 cm and an accuracy of 0.1 cm. The interviewers were trained by certified nutritionists to collect all measurements. Each measurement was repeated twice to ensure accuracy, and the average between the first and second measurement was taken. If the two readings differed by more than the previously established point (0.1 kg for weight, 0.5 cm for height, 0.5 cm for neck circumferences, and 1 cm for waist circumferences), a third measurement was taken. All three measurements were recorded, and the extreme value was excluded during data processing.

### 2.5. Data Management and Analysis Plan

NC was correlated with WC and BMI. In addition, we considered other variables of interest including age, socioeconomic status, educational level, region, department, and marital status. The numerical variables are presented with mean and standard deviation and the categorical variables as frequency and percentage. An ANOVA or *t*-test was used to evaluate differences between the mean of the anthropometric measurements by the variables of interest. The correlation between NC and other anthropometric measurements (BMI and WC) was evaluated with Pearson’s correlation coefficient. The following levels were used to assess the strength of correlation: insignificant (0.00–0.10), weak (0.10–0.39), moderate (0.40–0.69), strong (0.70–0.89), and very strong (0.90–1.00). ROC curves were used to evaluate the area under the curve (AUC) and the sensitivity (Se) and specificity (Sp) by sex according to the cut-off point established with the Youden Index, and thus evaluate NC as an indicator for an overweight or obesity in an individual according to BMI (overweight > 25, obese > 30) and WC (overweight: women > 80 cm; men > 95 cm; obese: men > 102; women > 88 cm). All analyses were performed with a significance level of 5%. The Stata SE v.16.1 program was used.

## 3. Results

### 3.1. General Characteristics of the Population

Table 1 shows the general characteristics of the population. Slightly more than half of the total population studied were women (52.2%), 37.7% were between 20 and 34 years of age; most were between middle and low socioeconomic status (42.8% and 41.8%, respectively), 61.2% had basic education, and most were married/cohabiting or single (47.7% and 42.4%, respectively). In most countries, the same trend was observed for all variables, except for educational level, where Peru shows a larger population with higher education.

### 3.2. Bivariate Analysis

Table 2 shows the bivariate and stratified analysis of mean NC, WC, and BMI. The means (SD) for NC, WC, and BMI were 35.6 (4.1), 88.3 (14.3), and 26.9 (5.6), respectively. For NC and WC, the measurements were higher in men (NC: 37.7 (0.1), WC: 89.2 (14.2)) than in women (NC: 33.7 (0.2); WC: 87.4 (14.4); *p* < 0.001). BMI was higher in women than in men ((27.5 (5.9), 26.3 (5.2); respectively, <0.001). As age increased, NC, WC, and BMI increased (*p* < 0.001). Although we found no difference by socioeconomic status in the total population for any of the measurements (NC, WC, and BMI), we observed that, in men, the WC and BMI decreased as the socioeconomic status increased but, in women, these measurements decreased. In almost all the categories of the variables evaluated, differences by sex were observed, except among the measurements of WC for those with low socioeconomic status, basic education, and BMI in the high school level and university or higher education.

### 3.3. Correlation

Table 3 shows the correlation between NC and WC with BMI. With respect to the total population correlation, the higher correlation was observed between NC and WC (r = 0.64) and between NC and BMI (r = 0.51). However, a moderate correlation was observed between NC and WC in men (r = 0.69) and a strong correlation in women (r = 0.70). Similarly, it was observed that the correlation increased between NC and BMI when stratified by sex (r = 0.65). When looking at the correlations by country, we can see that in Argentina, Brazil, and Peru, the correlation between NC and BMI increased when stratified by sex. In Brazil and Peru, the correlation between NC and WC was strong (r = 0.71, 0.72, and 0.70, respectively). When analyzed by sex, there was a strong correlation in men for all countries except Brazil and Costa Rica, and there was a strong correlation in women in Argentina, Chile, Peru, and Costa Rica. The correlation between NC and BMI was moderate in all countries; in the analysis by sex, there was a strong correlation in men in Chile, Peru, Costa Rica, and Colombia, whilst, in women, there was a strong correlation in Argentina, Chile, Peru, and Costa Rica. In all age groups, the correlations between NC and WC between NC and BMI were moderate. When analyzed by socioeconomic status, it was strong only for the low level, both in men and women. By educational level, there was only a strong correlation between NC and WC in men with basic and higher education (r = 0.72 and 0.70, respectively) and in women with basic education (r = 0.71). By marital status, the correlation between NC and WC was high in single men and women (r = 0.71 and 0.70, respectively).

### 3.4. Cut-Off, Se, Sp, and AUC

Table 4 shows the cut-off point established taking into account the values of the sensitivity and specificity analysis and AUC established by the ROC curve by sex and according to nutritional status as defined by BMI (overweight, BMI > 25; and obese, BMI > 30) and waist circumference (overweight: women > 80 cm; men > 95 cm; obese: men > 102; women > 88 cm). The AUC, compared to WC for overweight in men was 85.9 (CI 95% = 84.6–87.1) for the cut-off point of 39.0 cm NC (Se: 76.5%, Sp: 83.4%), whilst, for women, the AUC value was 88.3 (CI 95% = 87.0–89.7) for the cut-off point of 32.9 cm (Se: 77.4%, Sp: 77.6%). For obesity compared to WC, the AUC was 88.3 (CI 95% = 87.0–89.7) for the cut-off point of 39.8 cm (Se: 78.7%, Sp: 83.5%), and, in women, the AUC was 83.8 (CI 95% = 82.6–84.9), for the cut-off point of 33.7 cm (Se: 78.7%, Sp: 83.5%). Most countries showed very similar values, and Brazil showed the lowest cut-off points for both overweight and obesity in men and women (overweight: 37.4 cm in men and 32.0 cm in women and obesity: 38.5 cm in men and 32.9 cm in women). The AUC when compared with overweight and obesity values according to BMI are lower and the cut-off points are also lower. Thus, in the total population, the AUC for overweight in men is 82.5 (CI 95%: 81.3–83.7) for the cut-off point of 37.5 cm (Se: 73.6% and Sp: 78.0%) and in women, the AUC is 82.5 (CI 95%: 81.3–83.6) for the cut-off point of 33.1 cm (Se: 73.5% and Sp: 78.5%). In the case of obesity in men, the AUC is 83.0 (CI 95%: 81.4–84.7) for the cut-off point of 39.2 cm (Se: 75.9% and Sp: 79.9%), and, in women, the AUC is 84.4 (CI 95%: 83.2–85.7) for the cut-off point of 34.2 cm (Se: 80.0% and Sp: 75.9%).

## 4. Discussion

The aim of our study was to evaluate the correlation between NC and BMI and WC in Latin American populations living in urban areas and to propose cut-off points to determine overweight and obesity through Se and Sp analyses and AUC. The results showed a moderate correlation between CD and WC and NC and BMI in the total population studied, although the correlation was higher when the data were stratified. This correlation was slightly higher in women than in men, and the correlation was greater with WC than with BMI. We found that an NC cut-off point of 39 cm identifies overweight in men when compared to WC and 39.8 cm for obesity, and, in women, the cut off points were 32.9 and 33.7 cm, respectively. When compared with BMI, the cut-off point of NC for men was 37.5 cm and for obesity 39.2 cm, whilst in women, it was 33.1 and 34.2 cm, respectively.

The correlations we found in our study were moderate to strong. In a systematic review, a higher correlation was found between NC and WC (r = 0.85) and between NC and BMI (r = 0.88) [18]. This may be because this study was a review of 19 publications from Europe and Asia. In Asia, the body composition of its population is different to Latin Americans, and different BMI cut-off points are used to define overweight and obesity, which could explain some of the difference from the results of our study, where other cut-off values for BMI have been used to compare with NC, which differ from the highest cut-off points in South America; consequently, the body composition could be different and, therefore, the correlations could change. Brazil showed a moderate correlation; this was the lowest observed among all the countries, so it would be necessary to evaluate what leads to these values.

In our study, the correlations between NC and WC and between NC and BMI were stronger when stratified by sex. A similar situation has been described in an urban district in southern Israel, (men = 0.86; females: r = 0.85) [19] and in subjects with metabolic syndrome in Calcutta, India (males: r = 0.74 and females: r = 0.71) [20]. In Bangladesh, a moderate correlation was found, lower in women than in men (r = 0.46 vs. r = 0.61, respectively) [21]. A study conducted in Karachi, Pakistan, with students aged 18 to 20 years, found a strong correlation between NC and WC in men (r = 0.85) and a moderate correlation with women (r = 0.62) [14]. In Peru, a study conducted in a province of Lima showed a strong correlation in men and women (r = 0.74 and r = 0.72; respectively), slightly lower than that found in our study in Peru, but like that found in the general population [22].

With respect to the correlations between NC and BMI, a moderate correlation was found in our study. The author of the Peruvian study showed a strong correlation when stratified by sex (men: r = 0.72 and women r = 0.78) [22]. A study conducted in Brazil [23]) and another in Calcutta [19] both found a strong correlation in men (r = 0.73, r = 0.74, respectively) but a moderate correlation in women (r = 0.68 in both studies). These studies suggest that this may be because men have more muscle mass compared to women. However, it is worth mentioning that information differed from those we found in Brazil in our study, where the correlations were the lowest.

When analyzed by age, the correlations were moderate in our study. In Pakistan, a study conducted with university students between 18 and 20 years of age, the correlation between NC and BMI was strong in both men and women (r = 0.86; r = 0.70, respectively) [14]. In a study conducted in older adult Brazilian women, NC showed a moderate correlation with BMI (r = 0.67) and also with WC (r = 0.56) [24]. It is worth mentioning that the differences in the correlations found between NC, WC, and BMI compared to studies from Asian countries may be because the height and, in general, the body morphology in these populations are different.

Coutinho and collaborators in a study conducted with 2794 students between 6 and 19 years of age in Sao Paulo found a strong correlation between NC and BMI (r = 0.75) and WC (r = 0.81) [25]. Similar results were found when stratified by sex [26]. These differences in the results may be due to the fact that the populations of these studies were of different ages; therefore, the correlations could differ with age.

Regarding NC cutoff points, other studies have identified similar values to those found in our study. Thus, in Israel and a study conducted in Lima, Peru, the best NC cut-off point for overweight was found to be 37 cm for men and 34 cm for women [16,22]. In addition, the Lima, Peru, study identified that for obesity the cut-off point was 39.5 cm for men and 36.5 cm for women [21]. These values coincide with our results in the total population of our study, especially when the cut-off point was identified with the values for overweight and obesity for BMI.

Finally, to evaluate the advantages of using NC to assess overweight and obesity, it is necessary to understand the limitations of the other measurements applied in our study (WC and BMI) for comparison. Although we found a higher correlation between NC and WC than with BMI, the most used method to determine overweight and/or obesity is BMI. Several studies have discussed the accuracy of the use of the different anthropometric measurements that are most used in clinical practice and conclude that, in patients with overweight and obesity, the WC may present a greater margin of error in the measurement due to the difficulty of locating the anatomical reference points [27,28]. In addition, the WC requires more time and training and, like BMI, may be affected by the time of day when it is measured. Nevertheless, BMI uses universally accepted measures that can be performed by a relatively simple procedure (weight and height). Therefore, it remains the most useful measure for detecting overweight and obesity in medical practice, and it is more accurate when performed by trained personnel [27]. However, evidence shows that the existing anthropometric measurements are not entirely effective for adequately diagnosing overweight and/or obesity because of the difficulty in their application and accuracy in the measurement technique. It is for this reason that measurement of the NC has emerged as a simple, low-cost, and minimally invasive method. Likewise, it is not affected by the time of day when it is measured, nor does it change with food intake, nor is it sensitive to abdominal distension (16). Finally, it is not impaired by gas exchange on inhalation and exhalation. Therefore, it is considered an anthropometric measure that can be used but further research is required for its validation.

This study has some limitations, since the ELANS study did not take as exclusion criteria people who might have presented goiter related to iodine deficiency, so the measurement of NC could be affected. However, this proportion of the population is expected to be low. On the other hand, this study is only representative of the urban population of the countries under study and does not represent the population living in rural areas. Finally, it does not have information on chronic diseases: hypertension, diabetes, or others that could help to better identify the usefulness of the NC measure. Nevertheless, this is one of the few studies in Latin America that has a fairly large sample and has been carried out in different regions of each country. It also has different anthropometric measures that are rarely included in studies.

## 5. Conclusions

Our study found a moderate correlation between NC and WC and NC and BMI; both correlations improved when stratified by sex. The cutoff point of 39 cm identifies overweight men according to WC and 39.8 cm for obesity, and, in women, it was 32.9 and 33.7 cm, respectively. The cut-off point for NC in men, if considered overweight, determined by BMI, was 37.5 cm, and for obesity, 39.2 cm, while in women, it was 33.1 and 34.2 cm, respectively. Taking into account that the WC is a better indicator of central obesity, we consider that it is better to take into account the cut-off point that was found, taking into account this indicator. These results indicate that NC can be used as a simple, easy, and fast method to identify overweight and obesity in people and that it can be applied especially at the first level of care, since it does not require heavy equipment and can be easily transported. However, it is important to continue studying this tool as an anthropometric measurement so that it can be used in the different primary care health services. In addition, its potential as a predictor of different chronic diseases should be further investigated and compared with other anthropometric measures such as BMI and WC.

## Figures and Tables

**Table 1:** Sociodemographic characteristics of the Latin American population. Latin American Health and Nutrition Study/Estudio Latino Americano de Nutrición y Salud (ELANS).

Variables	Argentina	Brazil	Chile	Peru	Colombia	Costa Rica	Ecuador	Venezuela	Total
	n = 1266	n = 2000	n = 879	n = 1113	n = 1230	n = 798	n = 800	n = 1132	n = 9218
n (%)									
Sex									
Women	573 (54.7)	942 (52.9)	425 (51.7)	523 (53.0)	603 (51.0)	394 (50.6)	397 (50.4)	552 (51.2)	4409 (52.2)
Men	693 (45.3)	1058 (47.1)	454 (48.4)	590 (47.0)	627 (49.0)	404 (49.4)	403 (49.6)	580 (48.8)	4809 (47.8)
Age (years)									
15–19	152 (12.0)	235 (11.8)	118 (13.4)	165 (14.8)	148 (12.0)	121 (15.2)	128 (16.0)	156 (13.8)	1223 (13.3)
20–34	446 (35.2)	745 (37.3)	307 (34.9)	460 (41.3)	445 (36.2)	301 (37.7)	316 (39.5)	459 (40.6)	3479 (37.7)
35–49	379 (29.9)	608 (30.4)	252 (28.7)	294 (26.4)	335 (27.2)	224 (28.1)	222 (27.8)	313 (27.7)	2627 (28.5)
50–65	289 (22.8)	412 (20.6)	202 (23.0)	194 (17.4)	302 (24.6)	152 (19.1)	134 (16.8)	204 (18.0)	1889 (20.5)
Socioeconomic status									
High	65 (5.1)	705 (35.3)	80 (9.1)	225 (20.2)	67 (5.5)	108 (13.5)	104 (13.0)	62 (5.5)	1416 (15.4)
Medium	585 (46.2)	1034 (51.7)	388 (44.1)	355 (31.9)	384 (31.2)	428 (53.6)	582 (72.8)	190 (16.8)	3946 (42.8)
Low	616 (48.7)	261 (13.1)	411 (46.8)	533 (47.9)	779 (63.3)	262 (32.8)	114 (14.3)	880 (77.7)	3856 (41.8)
Education level									
Primary school	955 (75.4)	968 (48.4)	572 (65.1)	257 (23.1)	799 (65.0)	651 (81.6)	664 (83.0)	777 (68.6)	5643 (61.2)
High school	257 (20.3)	864 (43.2)	208 (23.7)	747 (67.1)	294(23.9)	101 (12.7)	84 (10.5)	142 (12.5)	2697 (29.3)
Undergraduate or higher	54 (4.3)	168 (8.4)	99 (11.3)	109 (9.8)	137 (11.1)	46 (5.8)	52 (6.5)	213 (18.8)	878 (9.5)
Marital status									
Single	441 (34.8)	852 (42.6)	398 (45.3)	443 (39.8)	570 (46.3)	359 (45.0)	315 (39.4)	534 (47.2)	3912 (42.4)
Marriage	634 (50.1)	929 (46.5)	406 (46.2)	587 (52.7)	562 (45.7)	368 (46.1)	414 (51.8)	493 (43.6)	4393 (47.7)
Separated, divorced, widowed	191 (15.1)	219 (11.0)	75 (8.5)	83 (7.5)	98 (8.0)	71 (8.9)	71 (8.9)	105 (9.3)	913 (9.9)

**Table 2:** Bivariate analysis for the total population and by sex between anthropometric measurements and variables of interest. Latin American Health and Nutrition Study/Estudio Latino Americano de Nutrición y Salud (ELANS).

	NC (Mean (SD))					WC (Mean (SD))					BMI (Mean (SD))				
	Total	*p* +	Male	Female	*p* ++	Total	*p* +	Male	Female	*p* ++	Total	*p* +	Male	Female	*p* ++
**Total**	35.6 (4.1)		37.7 (0.1)	33.7 (0.0)	<0.001	88.3 (14.3)		89.2 (14.2)	87.4 (14.4)	<0.001	26.9 (5.6)		26.3 (5.2)	27.5 (5.9)	<0.001
Country		<0.001					<0.001					<0.001			
Argentina	35.6 (4.0)		37.7 (0.2)	33.8 (0.1)	<0.001	88.5 (15.5)		90.2 (15.1)	87.1 (15.7)	<0.001	27.1 (6.0)		26.7 (5.4)	27.4 (6.4)	0.042
Brazil	34.8 (4.6)		36.8 (0.1)	32.9 (0.1)	<0.001	87.6 (14.7)		88.4 (14.6)	86.8 (14.8)	0.021	26.7 (5.7)		26.1 (5.4)	27.3 (5.9)	<0.001
Chile	37.3 (3.9)		39.5 (0.2)	35.2 (0.2)	<0.001	92.1 (14.3)		93.9 (13.4)	90.4 (14.9)	<0.001	28.1 (5.5)		27.6 (4.8)	28.5 (5.9)	0.009
Peru	35.4 (3.6)		37.5 (0.1)	33.5 (0.1)	<0.001	87.3 (12.3)		88.0 (12.4)	86.7 (12.1)	0.081	26.6 (4.9)		26.0 (4.7)	27.3 (5.1)	<0.001
Colombia	35.2 (3.5)		37.2 (0.1)	33.3 (0.1)	<0.001	85.0 (13.1)		86.3 (13.1)	83.7 (13.0)	<0.001	25.7 (5.0)		25.0 (4.7)	26.4 (5.3)	<0.001
Costa Rica	36.7 (3.9)		38.6 (0.2)	34.7 (0.2)	<0.001	91.9 (15.4)		91.9 (16.0)	91.9 (14.8)	0.978	27.6 (6.2)		26.6 (5.6)	28.7 (6.6)	<0.001
Ecuador	35.1 (3.7)		36.8 (0.2)	33.4 (0.2)	<0.001	87.4 (12.3)		87.3 (12.0)	87.5 (12.7)	0.794	26.8 (5.4)		25.7 (5.0)	27.8 (5.6)	<0.001
Venezuela	36.2 (4.2)		38.2 (0.2)	34.3 (0.1)	<0.001	88.8 (14.6)		90.0 (14.3)	87.7 (14.8)	0.010	27.3 (5.8)		26.9 (5.4)	27.6 (6.1)	0.031
Age (years)		<0.001					<0.001					<0.001			
15–19	33.8 (3.5)		35.2 (3.3)	32.0 (2.9)	<0.001	77.0 (11.4)		77.6 (11.1)	76.2 (11.6)	0.026	22.9 (4.5)		22.6 (4.2)	23.4 (4.9)	0.002
20–34	35.3 (3.9)		37.4 (3.5)	33.2 (3.1)	<0.001	85.6 (13.1)		87.3 (13.0)	83.9 (12.9)	<0.001	26.1 (5.2)		25.9 (5.0)	26.3 (5.4)	0.017
35–49	36.4 (4.2)		38.7 (3.9)	34.4 (3.4)	<0.001	92.3 (13.7)		93.9 (13.5)	90.8 (13.8)	<0.001	28.4 (5.6)		27.8 (5.2)	29.0 (5.9)	<0.001
50–65	36.4 (4.0)		38.7 (3.6)	34.7 (3.4)	<0.001	94.9 (13.3)		96.1 (12.6)	94.0 (13.8)	<0.001	28.8 (5.4)		27.8 (4.8)	29.5 (5.7)	<0.001
Socioeconomic status		0.269					0.280					0.579			
High	35.7 (4.2)		37.6 (4.0)	33.8 (3.5)	<0.001	88.7 (14.2)		90.6 (14.1)	86.6 (14.1)	<0.001	27.1 (5.5)		26.8 (5.4)	27.3 (5.7)	0.161
Medium	35.5 (4.2)		37.7 (3.8)	33.5 (3.4)	<0.001	88.4 (14.2)		89.6 (14.4)	87.2 (13.9)	<0.001	26.9 (5.5)		26.3 (5.2)	27.4 (5.7)	<0.001
Low	35.7 (3.9)		37.6 (3.7)	33.9 (3.3)	<0.001	88.0 (14.4)		88.3 (13.9)	87.7 (14.9)	0.240	26.9 (5.7)		26.0 (5.1)	27.7 (6.2)	<0.001
Educational level		0.012					<0.001					0.017			
Primary school	36.0 (4.1)		37.6 (3.9)	33.9 (3.4)	<0.001	88.7 (14.8)		88.9 (14.7)	88.5 (14.9)	0.316	27.0 (5.8)		26.1 (5.3)	27.9 (6.1)	<0.001
High school	35.0 (4.1)		37.6 (3.7)	33.4 (3.3)	<0.001	87.2 (13.5)		89.2 (13.4)	85.5 (13.3)	<0.001	26.7 (5.2)		26.4 (5.0)	26.9 (5.4)	0.019
Undergraduate or higher	36.0 (4.1)		38.2 (3.6)	33.7 (3.2)	<0.001	88.4 (13.4)		91.3 (12.6)	85.6 (13.6)	<0.001	27.0 (5.2)		27.1 (4.9)	26.9 (5.5)	0.543
Marital status		<0.001					<0.001					<0.001			
Single	35.0 (4.0)		36.9 (3.7)	33.1 (3.3)	<0.001	83.8 (13.9)		84.8 (13.5)	82.7 (14.2)	<0.001	25.3 (5.5)		24.9 (5.2)	25.8 (5.9)	<0.001
Marriage	36.0 (4.1)		38.4 (3.8)	34.1 (3.3)	<0.001	91.4 (13.6)		93.5 (13.3)	89.7 (13.7)	<0.001	28.0 (5.4)		27.6 (4.9)	28.4 (5.7)	<0.001
Separated, divorced, widowed	36.0 (4.1)		38.2 (3.9)	34.3 (3.5)	<0.001	92.0 (14.2)		93.8 (14.8)	91.1 (13.9)	0.008	28.3 (5.5)		27.6 (5.0)	28.6 (5.7)	0.008

+ *p* value: difference between categories of each variable; ++ *p* value: difference between sex inside each category of the variable; NC: neck circumference, WC: waist circumference, BMI: body mass index.

**Table 3:** Correlation between NC and WC or BMI by sex and variables of interest. Latin American Health and Nutrition Study/Estudio Latino Americano de Nutrición y Salud (ELANS).

	TOTAL		Men		Women	
	NC-WC	NC-BMI	NC-WC	NC-BMI	NC-WC	NC-BMI
Total	0.64	0.51	0.69	0.65	0.71	0.65
Country						
Argentina	0.71	0.56	0.73	0.60	0.80	0.77
Brazil	0.55	0.41	0.58	0.52	0.59	0.50
Chile	0.72	0.59	0.79	0.75	0.78	0.78
Peru	0.70	0.57	0.81	0.80	0.80	0.76
Colombia	0.65	0.50	0.75	0.73	0.68	0.68
Costa Rica	0.64	0.59	0.69	0.80	0.79	0.79
Ecuador	0.62	0.48	0.75	0.67	0.67	0.64
Venezuela	0.64	0.51	0.70	0.61	0.69	0.65
Age (years)						
15–19	0.61	0.50	0.63	0.61	0.68	0.62
20–34	0.63	0.50	0.67	0.62	0.67	0.62
35–49	0.64	0.48	0.68	0.62	0.68	0.63
50–65	0.59	0.42	0.66	0.55	0.63	0.60
Socioeconomic status						
High	0.61	0.49	0.62	0.56	0.63	0.58
Medium	0.64	0.50	0.70	0.64	0.68	0.63
Low	0.66	0.54	0.76	0.70	0.73	0.70
Educational level						
Primary school	0.64	0.51	0.72	0.66	0.71	0.67
High school	0.64	0.52	0.70	0.66	0.65	0.62
Undergraduate or higher	0.65	0.49	0.62	0.54	0.68	0.60
Marital status						
Single	0.65	0.54	0.71	0.67	0.70	0.65
Marriage	0.64	0.48	0.68	0.60	0.68	0.64
Separated, divorced, widowed	0.64	0.51	0.65	0.57	0.68	0.64

NC: neck circumference, WC: waist circumference, BMI: body mass index.

**Table 4:** Cut-off for NC, sensibility, specificity, and area under the curve compared by nutritional level of WC and BMI for the total population and by country. Latin American Health and Nutrition Study/Estudio Latino Americano de Nutrición y Salud (ELANS).

	Men				Women			
	Cut-Off for NC (cm)	Se (%)	Sp (%)	AUC (CI 95%) (%)	Cut-Off for NC (cm)	Se (%)	Sp (%)	AUC (CI 95%) (%)
TOTAL								
WC								
Overweight	39.0	76.5	83.4	85.9 (84.6–87.1)	32.9	77.4	77.6	83.8 (82.6–84.9)
Obesity	39.8	78.7	83.5	88.3 (87.0–89.7)	33.7	78.8	77.8	84.9 (83.8–86.0)
BMI								
Overweight	37.5	73.6	78.0	82.5 (81.3–83.7)	33.1	73.5	78.5	82.5 (81.3–83.6)
Obesity	39.2	75.9	79.9	83.0 (81.4–84.7)	34.2	80.0	75.9	84.4 (83.2–85.7)
Argentina								
WC								
Overweight	38.6	79.2	86.8	87.9 (84.6–91.2)	33.2	75.5	84.3	88.5 (86.0–90.9)
Obesity	39.0	89.4	79.6	90.6 (87.4–93.8)	33.6	84.2	78.7	89.6 (87.3–91.9)
BMI								
Overweight	38.0	64.6	82.5	76.6 (72.7–80.5)	33.4	76.3	82.5	87.5 (84.9–90.0)
Obesity	39.0	75.6	76.2	78.9 (73.5–84.3)	34.5	81.7	80.4	89.5 (86.9–92.0)
Brazil								
WC								
Overweight	37.4	74.6	70.0	78.5 (75.2–81.7)	32.0	75.0	63.2	75.9 (72.9–78.9)
Obesity	38.5	76.6	74.7	82.5 (78.9–86.1)	33.0	76.5	68.4	78.9 (76.2–81.6)
BMI								
Overweight	37.0	70.1	68.1	75.0 (71.9–78.1)	32.0	74.7	56.7	72.7 (69.7–75.7)
Obesity	37.1	77.8	63.6	76.1 (72.1–80.0)	33.0	77.7	57.8	76.1 (72.8–79.3)
Chile								
WC								
Overweight	39.4	77.0	82.2	88.4 (85.3–91.6)	33.5	84.4	76.9	87.9 (84.1–91.6)
Obesity	40.2	85.3	82.9	91.9 (89.1–94.7)	34.8	83.6	80.0	88.7 (85.6–91.7)
BMI								
Overweight	38.2	81.0	72.6	85.0 (81.2–88.8)	34.4	80.1	88.5	91.7 (89.1–94.3)
Obesity	40.2	85.7	82.2	90.4 (86.8–93.9)	35.3	86.4	76.4	89.3 (86.5–92.1)
Perú								
WC								
Overweight	38.6	84.1	86.5	91.7 (88.8–94.6)	32.7	76.9	87.8	89.4 (86.9–91.9)
Obesity	39.3	96.8	83.3	95.2 (93.4–97.0)	33.1	86.4	76.9	89.5 (87.0–91.9)
BMI								
Overweight	36.9	81.3	75.9	87.3 (84.4–90.3)	32.8	78.7	86.1	89.3 (86.7–91.9)
Obesity	39.1	92.0	84.0	92.6 (89.8–95.3)	33.8	90.3	71.3	88.1 (85.1–91.0)
Colombia								
WC								
Overweight	38.2	79.4	79.6	88.2 (85.3–91.1)	32.8	78.2	71.9	81.6 (78.2–84.9)
Obesity	39.9	77.5	86.4	89.3 (85.6–93.1)	33.8	88.0	58.8	84.1 (80.9–87.3)
BMI								
Overweight	37.0	83.8	71.5	86.3 (83.4–89.1)	32.5	82.7	64.7	81.3 (78.0–84.7)
Obesity	39.1	83.1	82.9	89.9(86.4–93.5)	33.8	86.6	73.0	87.4 (84.2–90.5)
Costa Rica								
WC								
Overweight	38.9	87.3	79.2	86.9 (83.2–90.7)	32.6	84.7	81.9	90.5 (87.2–93.9)
Obesity	39.6	81.8	76.8	87.2 (82.9–91.5)	33.5	89.0	72.5	89.1 (86.0–92.3)
BMI								
Overweight	38.4	79.9	87.1	90.2 (87.2–93.2)	33.7	81.1	82.2	91.0 (88.1–93.9)
Obesity	39.6	85.6	78.3	89.1 (85.2–93.1)	34.8	94.0	59.7	88.9 (85.6–92.1)
Ecuador								
WC								
Overweight	37.5	82.9	73.8	85.5 (81.4–89.7)	32.9	74.7	82.0	86.2 (82.5–89.9)
Obesity	38.7	87.5	77.9	87.7 (82.2–93.1)	33.7	69.7	79.3	82.2 (78.1–86.2)
BMI								
Overweight	36.5	82.6	74.7	86.3 (82.8–89.9)	32.9	74.2	73.4	79.9 (75.4–84.5)
Obesity	38.7	75.0	80.0	83.8 (78.7–88.9)	34.2	79.0	83.2	84.6 (80.3–88.9)
Venezuela								
WC								
Overweight	39.0	80.5	81.9	86.4 (83.0–89.8)	33.1	76.1	82.8	86.6 (83.4–89.8)
Obesity	39.8	82.0	80.3	87.7 (83.9–91.4)	33.7	83.8	77.2	86.7 (83.7–89.7)
BMI								
Overweight	37.6	78.3	79.1	84.6 (81.3–88.0)	33.3	77.1	79.3	84.3 (81.0–87.6)
Obesity	39.8	73.3	81.1	80.1 (75.4–84.9)	34.4	82.4	75.9	83.7 (80.1–87.3)

NC: neck circumference, WC: waist circumference, BMI: body mass index.

## Data Availability

No new data were created or analyzed in this study. Data sharing is not applicable to this article.
